# What’s lurking within: When retroperitoneal calciphylaxis goes off-skin

**DOI:** 10.1016/j.radcr.2026.07.120

**Published:** 2026-08-26

**Authors:** Hannah Cho, Dillon Sommer, Bryant Tran, Ozivefueshe Dimowo, Ryan O’Connell, Uttam Reddy, Roozbeh Houshyar

**Affiliations:** a Irvine Department of Radiological Sciences, University of California, 101 The City Drive South, Orange, CA 92868, USA; b Irvine Department of Pathology and Laboratory Medicine, University of California, 101 The City Drive South, Orange, CA 92868, USA; c Irvine Department of Nephrology, University of California, 101 The City Drive South, Orange, CA 92868, USA

**Keywords:** Visceral calciphylaxis, Retroperitoneal, Case report, Renal, Computed tomography

## Abstract

We present a rare case of retroperitoneal calciphylaxis in a 63-year-old woman with end-stage renal disease secondary to hypertension. The disease involved bilateral retroperitoneum and was incidentally found on CT imaging performed for renal transplant evaluation. Imaging revealed diffuse soft tissue hyperattenuation, calcified nodules and cortical calcifications. Subsequent biopsy of the perinephric fat demonstrated fat necrosis and vascular wall calcification, consistent with calciphylaxis. The patient was medically cleared for renal transplantation and has shown improved kidney function on follow-up. While both cutaneous and visceral calciphylaxis typically carry poor prognoses, this case represents an indolent visceral variant discovered in the absence of cutaneous or systemic complications.

## Introduction

Calciphylaxis is a rare, life-threatening disorder caused by calcification of small and medium-sized vessels, most often in the skin [1]. It leads to ischemia, painful lesions, necrosis and carries a high mortality rate of 40%-80% [1] largely due to infection and impaired wound healing. The condition is strongly associated with end-stage renal disease (ESRD) on hemodialysis, with the reported incidence around 3.5% [2], where abnormalities in calcium-phosphate metabolism promote vascular calcification. Reported risk factors include obesity (BMI > 30 kg/m^2^), diabetes mellitus, and hyperphosphatemia [1].

Diagnosis can be challenging due to clinical manifestations varying widely depending on the disease stage and location [1]. While painful skin plaques or ulcers often raise clinical suspicion, biopsy remains the gold standard, typically demonstrating vascular intimal calcification, microthrombi, and luminal narrowing. However, biopsy carries risks, and findings may be nonspecific, showing subtle early changes to frank necrosis [1]. Imaging modalities such as CT, bone scintigraphy, and mammography can serve as valuable adjuncts by revealing areas of vascular calcification or soft tissue calcification [3,4].

Although calciphylaxis most commonly presents in cutaneous form, visceral calciphylaxis is uncommon and likely an underrecognized variant [5]. It has been reported to involve organs such as the lungs, gastrointestinal tract, and mesentary [5,6]. However, to our knowledge, there are no prior reports describing isolated retroperitoneal calciphylaxis without cutaneous involvement. This case expands the spectrum of visceral disease and highlights the importance of recognizing atypical sites of vascular calcification on imaging.

## Case report

A 63-year-old woman with end-stage renal disease (ESRD) secondary to hypertension was evaluated for kidney transplantation. She had been receiving hemodialysis for 13 years and a history of secondary hyperparathyroidism requiring subsequent parathyroidectomy. Her initial transplant evaluation was initially in 2018 but was delayed until 2021 due to insurance-related issues.

At presentation, the patient was clinically well with no apparent physical or systemic symptoms or cutaneous manifestations of calciphylaxis. Physical examination demonstrated no painful skin lesions, retiform purpura, livedo, ulceration, or necrosis. Laboratory values were significant for blood urea nitrogen (BUN) of 33 mg/dL, creatinine 5 mg/dL, and estimated glomerular filtration rate of 9 mL/min/1.73 m^2^. Serum calcium was mildly decreased at 8 mg/dL.

**Fig. 1:**
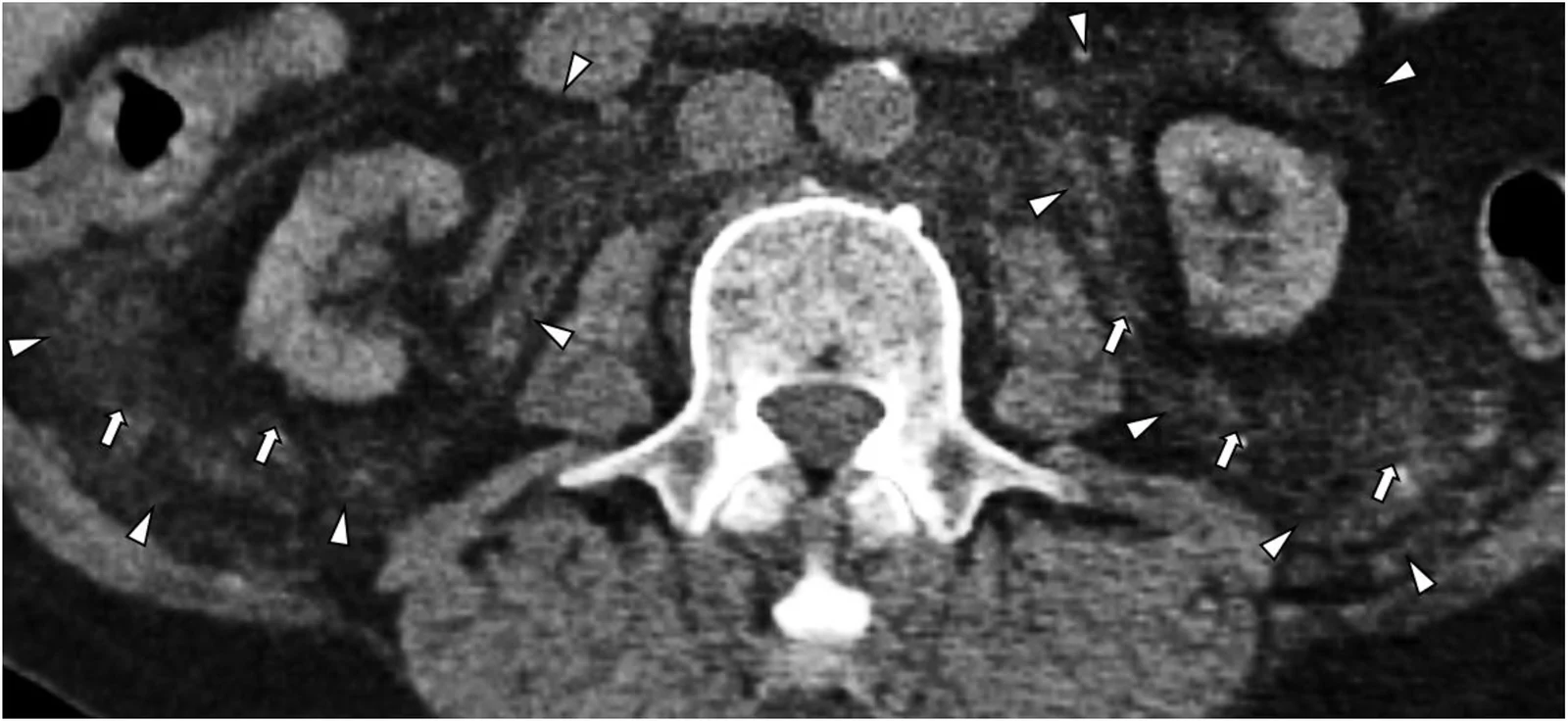
Axial noncontrast CT of the abdomen demonstrating bilaterally atrophic kidneys and diffuse amorphous hyperattenuating haziness and ill-defined nodularity throughout the bilateral retroperitoneal perinephric compartments (white arrowheads) with subtle areas of punctate calcification (white arrows) and relative sparing of the immediate perinephric fat.

As part of routine pretransplant evaluation, a noncontrast CT of the abdomen and pelvis was obtained and compared with a prior examination performed approximately six years earlier. The kidneys demonstrated progressive bilateral atrophy with cortical hyperattenuation, cortical calcifications, and multiple simple and complex renal cysts. A new 5-mm exophytic isodense lesion was also identified within the upper pole of the right kidney. Additionally, there was interval development of diffuse amorphous hyperattenuation throughout the bilateral retroperitoneum involving the parietal peritoneum, anterior and posterior pararenal fascia, lateroconal fasci, and anterior pararenal spaces, with relative sparing of the immediate perinephric fat (Fig. 1). Numerous partially calcified soft tissue nodules were distributed throughout the involved retroperitoneal fat. These findings were absent or substantially less conspicuous on the prior CT examination. Given the progressive infiltrative appearance, differential considerations included infiltrative malignancy, inflammatory disorders, and unusual metabolic deposition processes, prompting referral to hematology and image-guided biopsy.

**Fig. 2:**
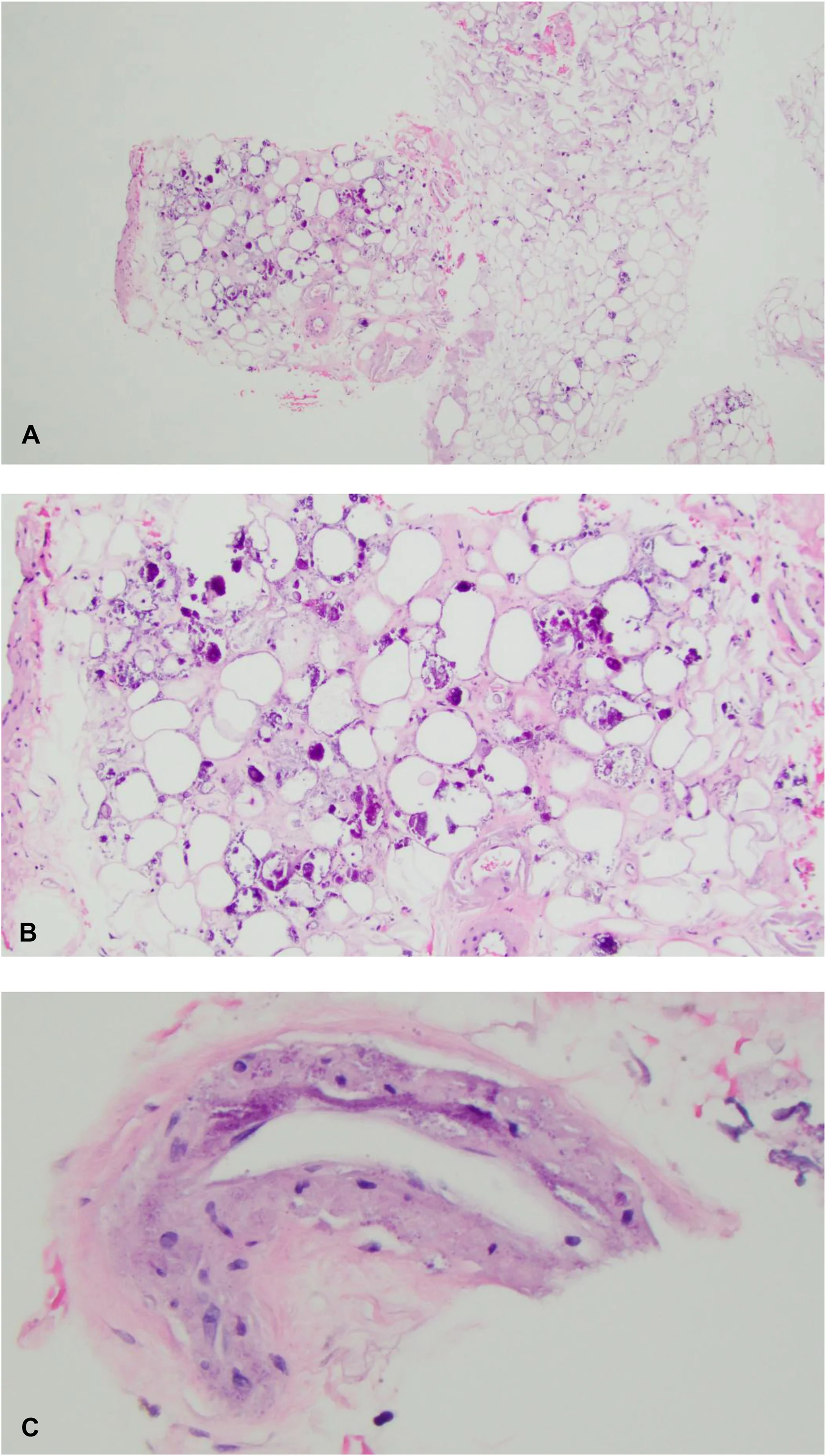
(A and B) H&E stained tissue section shown at 100× magnification of the left pararenal soft tissue biopsy showing fibroadipose and vascular tissue with abundant granular basophilic calcifications. (C) Early calcifications can be seen within the vessel walls at 400× magnification.

CT-guided core biopsy of the left perinephric fat was performed, and histopathologic examination demonstrated mature adipose tissue with fat necrosis and granular basophilic calcification involving small vessel walls, findings consistent with calciphylaxis (Fig. 2). No evidence of malignancy was identified. Given the absence of cutaneous or systemic symptoms, no calciphylaxis-directed therapy was initiated. The patient’s postbiopsy course was notable for transient asymptomatic hyperkalemia requiring urgent hemodialysis after which the patient recovered without further complication.

After malignancy was excluded, she underwent renal transplantation. Ten months after transplant, renal allograft function remained stable and remained free of cutaneous manifestations of clinical progression of calciphylaxis.

## Discussion

Calciphylaxis is most often described as a cutaneous disease process characterized by medial calcification of small- and medium-sized vessels, leading to ischemia, necrosis, and a mortality rate up to 80% [7]. Although classically associated with painful skin lesions, the clinical presentation is heterogenous and patients may not develop the prototypical necrotic lesions, suggesting that calciphylaxis can manifest in more subtle or atypical ways [8]. Increasing recognition of visceral calciphylaxis has expanded the disease spectrum beyond the skin, with reported involvement most frequently affecting the mesentery and gastrointestinal tract [6,9,10]. These cases typically present with abdominal pain, gastrointestinal bleeding, bowel ischemia or perforation [11]. Furthermore, most published cases of visceral calciphylaxis occur in conjunction with cutaneous disease, whereas isolated extracutaneous involvement is exceptionally uncommon [5,12].

Our case illustrates a rare presentation of isolated retroperitoneal calciphylaxis involving bilateral perinephric spaces without any cutaneous manifestations. To our knowledge, this variant has not been reported in the literature. The diagnosis was complicated by the absence of classic skin lesions, lack of pain, and incidental discovery on imaging. While biopsy remains the gold standard for diagnosis, its invasive nature limits routine use, and radiology offers a valuable noninvasive diagnostic tool, particularly when disease manifests atypically [4,13]. The patient’s history of long-term dialysis, obesity, female sex, prior hyperparathyroidism, and imaging findings offered strong clinical context for calciphylaxis despite the atypical presentation [14,15].

Similar to cutaneous calciphylaxis, which carries a high mortality rate due to the risk of superinfection and sepsis, visceral variants have been reported to have a poor prognostic course [5,16]. The true mortality rate of isolated visceral calciphylaxis remains uncertain, but most reported cases have fatal outcomes due to complications including sepsis, bleeding, and complications from coexisting cutaneous lesions [6,10,17,18]. In contrast, our patient successfully underwent kidney transplantation and remained clinically stable with intact kidney function, no development of cutaneous manifestations, and no evidence of progression of calciphylaxis. This case contributes to the limited literature on retroperitoneal calciphylaxis and underscores the need for radiologists to consider this diagnosis in dialysis patients with progressive bilateral retroperitoneal calcifications and infiltrative soft tissue abnormalities, particularly in those with longstanding dialysis and other risk factors [11].

## Conclusion

This case highlights a rare variant of calciphylaxis localized to the retroperitoneal region and discovered incidentally on CT. Unlike the classic cutaneous form, which often presents acute and carries a high mortality risk, this case followed an indolent course without cutaneous involvement or complications. Recognition of such atypical presentations broadens the clinical and radiologic understanding of calciphylaxis and underscores the importance of considering visceral disease in dialysis patients with unexplained retroperitoneal calcifications.

## Patient consent

Written informed consent was obtained from the patient for publication of this case report.
